# Body conformation traits in early-lactation associated with clinical mastitis and lameness in lactating Chinese holstein cows

**DOI:** 10.1186/s12917-024-03931-1

**Published:** 2024-03-08

**Authors:** Zhipeng Zhang, Jiayu Yang, Yiyang Yao, Dasheng Wang, Xubin Lu, Zhangping Yang

**Affiliations:** 1 College of Animal Science and Technology, Yangzhou University, Yangzhou, 225009 P. R. China; 2 Joint International Research Laboratory of Agriculture and Agri-product Safety of Ministry of Education of China, Yangzhou University, Yangzhou, 225009 P. R. China

**Keywords:** Dairy cow, Body conformation traits, Linear type score, Mastitis, Lameness

## Abstract

**Background:**

Comprehending the correlation between body conformation traits of cows at the early stages of lactation and prevalent lactation diseases might facilitate the execution of selection and feeding strategies that prioritize cow health. This study aimed to evaluate the impact of body conformation traits on the incidence of clinical mastitis and lameness in Chinese Holstein cows. From a pasture herd of 1472 early lactating Chinese Holstein cows, we evaluated 20 body conformation traits. During lactation, this pasture herd was visited weekly to gather clinical mastitis and lameness data. A nine-point scale was used to determine the conformation traits of cows to clarify their linear characters, including frame capacity, rump (RU), feet and leg (FL), mammary system (MS), and dairy character. A longitudinal binary disease (0 = healthy; 1 = diseased) data structure was created by allocating disease records to adjacent official test dates. The impact of body conformation traits on the risk of developing diseases (clinical mastitis and lameness) was analyzed using the logistic regression models.

**Results:**

Compared to cows with low total scores (75–79 points), those with high total scores (80–85 points) of body conformation traits had a significantly lower risk of mastitis (*P* < 0.001). The disease status (0 or 1: binary variable) of clinical mastitis in lactating cows was significantly impacted negatively by age (*P* < 0.05). The fore udder attachment (FUA), angularity, rear attachment height (RAH), and rear teat placement (RTP) were all significantly associated with clinical mastitis during lactation (*P* < 0.05). The rear leg-rear view (RLRV) was significantly correlated with correlated considerably (*P* < 0.05) with lameness during lactation. An ideal score of four points on the lameness risk dimension of the RLRV may indicate a low risk of lameness. Since the risk of mastitis decreased as this trait score increased, the RTP may be an ideal marker for mastitis risk.

**Conclusions:**

According to the study, clinical mastitis and lameness risks in cows can be estimated using their body conformation traits. Cows with more centrally located rear teats have a lower risk of mastitis. These results may help dairy farmers identify cows at high risk of disease early in lactation and aid in breeding for disease resistance in cows.

## Background

Body conformation traits in dairy herd selection indexes are widely acknowledged and are one of the initial non-productive trait evaluations [1]. The linear type score is an active standard for accurately assessing the body conformation traits of dairy cattle [2] after identification. This method rates the ideal performance by quantifying the actual performance state of body conformation traits and linearly arranging the degree of change from one biological extreme to another [3]. Usually referred to as an early predictor of functional traits, it reduces the decline in cow fertility and health [4].

The immune system of early lactation cows (0–70 days in milk) is weakened for several reasons, including nutritional drainage [5], calving-related stress [6], metabolic disorders [5], management decisions [7], housing [8], and other factors. This may increase the risk of disease during lactation. The ’health of the cows during lactation has a direct impact on the quality of the milk. Therefore, it is crucial for farm management to protect the health of lactating cows. The two most common diseases in lactating cows that result in significant economic loss for the farm are mastitis and lameness [9, 10]. In a previous study, mastitis and lameness, which are binary traits, had an overall incidence of 17% and 16%, respectively [11]. Several factors, such as bacterial infection, mechanical damage, and genetics, influence disease occurrence in dairy cows. The cow’s conformation is crucial in disease onset, which is equally important. For example, among the conformation traits of the body, the rump traits are associated with health disorders such as dystocia [12]. However, to the best of our knowledge, there are very few studies on how Chinese Holstein cow’s clinical mastitis and lameness are affected by body conformation. More importantly, the association between body conformation traits in early lactation and clinical mastitis and lameness in dairy cows has not been analyzed to date. However, such analysis would undoubtedly provide valuable insights for farmers.

There are over 14 million dairy cows in China. However, the broader global applicability of this research is a factor worth considering. Most of China’s imported Holstein dairy cattle were produced by cross-breeding local yellow cattle and imported Holstein bulls, originally imported from Europe and North America [13]. Genetically, the current Chinese Holstein population is similar to other global Holstein populations [14]. The degree of linkage disequilibrium was comparable in the Chinese and Nordic Holstein populations, and there was a strong correlation (*r* = 0.97) between the two populations [13]. Although the study results are essential in the local context, it is important to consider how these findings and insights could broaden the knowledge base of farmers and scientists dealing with similar issues in other regions and countries.

This study aimed to evaluate the impact of body conformation traits on the susceptibility to common conditions (clinical mastitis and lameness) in Chinese Holstein cows to understand better the relationship between early-lactation cow body conformation traits and common lactation diseases. Additionally, it can help with the execution of selection that prioritize cow health. This could be relevant for farmers in different countries.

## Results

### Descriptive statistics of body conformation traits

The variation values of the 20 linear scores for body conformation features ranged from 3.02 ± 1.20 (SRL) to 7.88 ± 0.98 (ST), as shown in Table 1. The traits of the body conformation mean total score (TS) value was 80.29, while the mean values of the traits in the six components ranged from 70.65 ± 3.17 (RU) to 86.06 ± 2.01 (FC). The differences from the theoretical optimal score ranged from − 0.02 (UD) to 4.24 (CW), and the CV was > 8.75%. The difference between the position score and theoretical optimal score went from 13.94 (FC) to 29.35 (RU), and the CV was > 2.34%.

**Table 1:** Descriptive statistics for body conformation traits in Chinese holstein cows

Trait	Mean	SD	CV (%)	Ideal Scores	Optimal Gap
**Frame capacity (FC)**	86.06	2.01	2.34	100	13.94
Stature (ST)	7.88	0.98	12.44	8	0.12
Chest width (CW)	4.76	0.84	17.65	9	4.24
Body depth (BD)	6.63	0.58	8.75	7	0.37
Loin strength (LS)	7.06	0.73	10.34	9	1.94
**Rump (RU)**	70.65	3.17	4.49	100	29.35
Pin setting (PS)	4.80	1.07	22.29	5	0.2
Pin width (PW)	6.71	0.68	10.13	9	2.29
**Feet and legs (FL)**	83.26	3.49	4.19	100	16.74
Feet angle (FA)	5.85	0.96	16.41	7	1.15
Heel depth (HD)	5.71	0.95	16.64	9	3.29
Bone quality (BQ)	6.39	0.79	12.36	9	2.61
Set of rear legs (SRL)	3.02	1.20	39.74	5	1.98
Rear leg-rear view (RLRV)	6.90	1.14	16.52	9	2.1
**Mammary system (MS)**	82.19	2.67	3.25	100	17.81
Udder depth (UD)	5.02	1.09	21.71	5	-0.02
Median suspensory (MS)	4.99	1.04	20.84	9	4.01
Fore udder attachment (FUA)	5.72	0.83	14.51	9	3.28
Fore teat placement (FTP)	5.06	0.48	9.49	6	0.94
Fore udder length (FUL)	4.61	0.79	17.14	5	0.39
Rear attachment height (RAH)	6.32	0.82	12.97	9	2.68
Rear attachment width (RAW)	6.17	1.05	17.02	9	2.83
Rear teat placement (RTP)	5.71	0.78	13.66	6	0.29
**Dairy character (DC)**	82.04	2.26	2.75	100	17.96
Angularity (ANG)	5.83	0.67	11.49	9	3.17
**Total score (TS)**	80.29	1.99	2.48	100	19.71

Ideal score: the most desirable score for each conformation trait; optimal gap: the difference between the mean and the ideal score for each conformation trait. The ST, CW, BD, PS, PW, FA, HD, SRL, UD, MS, FUL, RAH and RAW traits were scored linearly after measurement. The scores of LS, BQ, RLRV, FUA, FTP and RTP traits were scored subjectively. The FC, RU, FL, MS, DC and TS are composite traits

### Incidence of disease in cows with different body conformation traits

Using the TS of body conformation traits, cows were classified into two groups based on industry standards: excellent (TS ≥ 80 points) and general (TS < 80 points). Table 2 displayed the incidence of mastitis and lameness in the various groups. From the perspective of TS, the incidence of clinical mastitis and lameness in the excellent group of cows decreased by 39.44% and 14.17%, respectively, compared to the general group. The incidence of clinical mastitis of cows in the excellent group was significantly lower than that in the general group (*P* < 0.001). We calculated the incidence of mastitis in cows with nine different MS traits or ANG and the incidence of lameness in cows with five other FL traits to further explore the differential detail in the incidence of mastitis and claudication induced by different TS of body conformation traits (Table 3). The findings showed that the cows with mastitis were found in nearly every linear score class of the nine MS traits and ANG. When the score grade increased in terms of rear teat placement (RTP), the incidence of mastitis exhibited a decreasing trend. A small range of scoring grades contained the majority of the distribution of lame cows in each of the five FL traits. Furthermore, there was no consistent and regular relationship between the five FL traits and the incidence of lameness.

**Table 2:** Incidence of clinical mastitis and lameness in Chinese holstein cows with different total score grades of body conformation traits

Diseases	Excellent group (TS ≥ 80 points)		General group (TS < 80 points)		Drop (%)	X^2^	***P*** value
	N°	Incidence (%)	N°	Incidence (%)			
**Mastitis**	188	37.45	220	22.68	39.44	36.02	< 0.001
**Total**	502	—	970	—	—		
**Lameness**	12	2.40	20	2.06	14.17	0.17	0.684
**Total**	502	—	970	—	—		

**Table 3:** Incidence of clinical mastitis and lameness in Chinese holstein cows with different body conformation traits and different scores (1 to 9 points)

Trait	1	2	3	4	5	6	7	8	9
	**Incidence of mastitis (%)**								
**UD**	—	33.33	42.11	32.12	26.98	24.8	12.82	14.29	100.00
**MS**	—	0.00	31.03	35.22	25.62	22.37	20.69	11.11	—
**FUA**	—	0.00	60.00	40.00	34.33	24.62	19.77	42.86	—
**FTP**	—	—	60.00	22.50	29.02	18.07	20.00	100.00	—
**FUL**	—	—	11.11	31.65	25.38	15.49	40.00	0.00	—
**RAH**	—	—	0.00	20.83	16.90	27.76	31.10	15.38	0.00
**RAW**	100.00	50.00	38.46	25.81	23.47	34.07	22.57	24.14	—
**RTP**	—	—	—	40.00	34.02	26.72	13.49	0.00	—
**ANG**	—	—	100.00	28.57	38.21	24.76	16.16	50.00	—
	**Incidence of lameness (%)**								
**FA**	0.00	0.00	0.00	3.33	0.74	3.04	1.38	0.00	—
**HD**	—	0.00	11.11	12.50	2.11	2.86	0.00	0.00	—
**BQ**	—	0.00	0.00	0.00	0.00	1.53	3.11	4.00	—
**SRL**	7.41	1.59	3.16	0.74	2.38	0.00	0.00	0.00	0.00
**RLRV**	—	0.00	0.00	0.00	3.45	1.88	2.63	2.02	0.00

The body conformation traits described in the table are only the MS traits associated with the site of mastitis and the FL traits associated with the site of lameness

### Effects of total score of body constitution traits and age on the risk of disease

Table 4 shows that the incidence of mastitis in lactation cows was significantly influenced by the total score of body conformation traits in early lactation (*P* < 0.001). In contrast, the incidence of lameness was not significantly affected by age (*P* > 0.05). The incidence of mastitis in lactating cows was significantly impacted by age (*P* < 0.05), although the incidence of lameness (*P* > 0.05) was not significantly affected by age. According to logistic regression results, the risk of mastitis and lameness increases with cow age. Additionally, the total score of body conformation traits for cows in the excellent group was 0.49 and 0.86 times lower, respectively, than the risk of mastitis and lameness in the general group.

**Table 4:** Effect of age (years) and the total score (points) of body conformation traits on the risk of clinical mastitis and lameness in Chinese holstein cows

Diseases	Effect	Level	B	SE	Wald χ^2^	df	OR value	95% confidence interval		***P*** value
								Lower limit	Upper limit	
**Mastitis**	Age	3 vs. 2	1.13	0.26	18.51	1	3.56	2.05	6.17	< 0.001
		4 vs. 2	1.38	0.27	25.85	1	4.34	2.47	7.64	< 0.001
		5 vs. 2	2.03	0.81	6.36	1	8.00	1.61	39.80	0.012
	TS	80–85 vs. 76–79	-0.71	0.17	17.69	1	0.49	0.35	0.68	< 0.001
**lameness**	Age	4 vs. 3	0.19	0.52	0.13	1	1.26	0.44	3.62	0.665
	TS	80–85 vs. 76–79	-0.62	1.05	0.34	1	0.86	0.31	2.39	0.766

B: unstandardized regression weight; SE: standard error for B; Wald χ^2^: the test statistic for the individual predictor variable; df: degrees of freedom. For mastitis, the 2 years was the base class, while 76–79 TS (points) of the body conformation traits points for age. For lameness, the 3 years and 76–79 points were the base classes of the age and TS of the body conformation traits

### Effect of mammary system traits on the risk of mastitis

We divided all traits into high and low groups following industry standards using the median value as the boundary because the mastitis-affected cows were dispersed throughout various grades of the nine MS traits and ANG. The group with high scores for the traits fore udder attachment (FUA), ANG, and RTP had a low risk of onset, while the group with low scores for the rear attachment height (RAH) traits had a high risk of onset, as indicated in Table 5.

**Table 5:** Effect of the mammary system traits on the risk of clinical mastitis in Chinese holstein cows

Trait	Level (points)	B	SE	Wald χ2	df	OR value	95% confidence interval		***P*** value
							Lower limit	Upper limit	
**UD**	6–9 vs. 2–5	-0.15	0.22	0.50	1	0.86	0.56	1.31	0.480
**MS**	6–8 vs. 2–5	-0.07	0.20	0.12	1	0.93	0.63	1.39	0.732
**FUA**	6–8 vs. 2–5	-0.49	0.19	6.79	1	0.61	0.43	0.89	0.009
**FTP**	6–8 vs. 3–5	-0.24	0.31	0.58	1	0.79	0.43	1.45	0.445
**FUL**	6–8 vs. 3–5	-0.40	0.31	1.68	1	0.67	0.37	1.22	0.195
**RAH**	6–9 vs. 3–5	0.751	0.31	6.05	1	2.12	1.17	3.86	0.014
**RAW**	5–8 vs. 1–4	-0.31	0.35	0.75	1	0.74	0.37	1.47	0.386
**RTP**	7–8 vs. 4–6	-0.92	0.29	10.43	1	0.40	0.23	0.70	0.001
**ANG**	6–8 vs. 3–5	-0.39	0.20	3.91	1	0.68	0.46	1.00	0.048

B: unstandardized regression weight; SE: standard error for B; Wald χ^2^: the test statistic for the individual predictor variable; df: degrees of freedom. According to the industry standard, the linear scoring median value of the trait was defined as the boundary, higher than the median value was defined as the group with high level of the trait, lower than the median value was defined as the group with low level of the trait, and the level of the low group was taken as the reference level of the trait effect

### Effect of feet and leg traits on the risk of lameness

Based on industry standards, it is illogical to classify the cows with lameness as high or low because they were clustered above or below the median score for the five FL traits. As a result, we determined the lowest score for each trait based on the scores of the five FL traits in cows (Table 6) to examine the effect of traits on lameness. We discovered that the rear leg-rear view (RLRV) considerably impacted the prevalence of lameness in cows. In cows with a linear score of four points for the RLRV, which was 40% higher than in cows with a linear score of 1, the risk of lameness was shown to be the lowest.

**Table 6:** Effect of the Feet and leg traits on the risk of lameness in Chinese holstein cows

Trait	Level (points)	B	SE	Wald χ2	df	OR value	95% confidence interval		*P* value
							Lower limit	Upper limit	
**FA**	5 vs. 4	-1.87	1.61	1.36	1	0.15	0.01	3.58	0.244
	6 vs. 4	-0.25	1.41	0.03	1	0.78	0.05	12.28	0.858
	7 vs. 4	-0.61	1.53	0.16	1	0.54	0.03	10.99	0.691
**HD**	4 vs. 3	-1.23	2.09	0.35	1	0.29	0.01	17.55	0.557
	5 vs. 3	-1.54	1.94	0.63	1	0.22	0.01	9.59	0.428
	6 vs. 3	-1.60	1.79	0.79	1	0.20	0.01	6.80	0.373
**BQ**	7 vs. 6	1.12	0.69	2.65	1	3.08	0.80	11.89	0.103
	8 vs. 6	1.54	1.70	0.82	1	4.67	0.17	129.94	0.364
**SRL**	2 vs. 1	-2.36	0.98	5.78	1	0.10	0.01	0.65	0.016
	3 vs. 1	-2.04	0.99	4.31	1	0.13	0.02	0.89	0.038
	4 vs. 1	-3.14	1.37	5.24	1	0.04	0.00	0.64	0.022
	5 vs. 1	-2.51	1.73	2.11	1	0.08	0.00	2.40	0.146
**RLRV**	6 vs. 5	-1.34	1.31	1.04	1	0.26	0.02	3.43	0.308
	7 vs. 5	-1.37	1.21	1.28	1	0.25	0.02	2.74	0.259
	8 vs. 5	-2.16	1.30	2.76	1	0.12	0.01	1.47	0.097

B: unstandardized regression weight; SE: standard error for B; Wald χ^2^: the test statistic for the individual predictor variable; df: degrees of freedom. The reference level of each individual conformational feature is determined by the minimum score within the group

## Discussion

In earlier studies [3, 15–17], the disease was hardly mentioned. The research on the association between body conformation traits and reproductive performance noted that body conformation traits impact reproductive diseases [18]. To the best of our knowledge, no reports have been made of other standard and recurrent ailments in dairy cows, such as mastitis and lameness. We are the first to document how the likelihood of mastitis and lameness in Chinese Holstein cows is affected by linear scores for body conformation traits determined by the most recent industry standards. We discovered that mastitis and lameness were less common in cows with 80–85 points of TS of body conformation traits than in cows with 75–79 points of TS. A body conformation trait with a higher TS indicates a more symmetrical body conformation. According to Stefánsdóttir et al. (2017) and de Almeida et al. (2019), this could be a physiologically healthy state that increases resilience to disease. The incidence of mastitis was significantly influenced by the TS of body conformation traits in this study, although lameness was not significantly impacted. We suppose there are two explanations for this. On the one hand, the MS and ANG traits had score weights roughly twice as high as the FL traits. Because there were more individuals with mastitis than lameness in a single farm, there was more excellent space for the incidence of mastitis to decrease than that of lameness. However, it has the advantage that random factors, beyond the cow effect, are intrinsically compensated. The TS for conformation traits in this study is a composite trait for type traits. It is a collection of linear traits weighted following the breeding goals of each country, such as frame, dairy strength, udder, feet, and legs [19]. The confirmation of this may necessitate a larger sample size and more farms, which could pose limitations on studies investigating body conformation trait factors associated with lameness.

The ages of the cows in this study with detected body conformation traits ranged from 2 to 5 years. As we are all aware, animal morphological development increases with age. Therefore, the age of a cow may have an impact on its body conformation traits. There was less morphological variance because the cows in this study may have grown to their full size. According to much earlier research, age has a minimal impact on body conformation traits [20]. For instance, body length, rump height, navel height, body depth, flank circumference, and chest circumference were unaffected by the cow’s age [21]. The current study also considered how age affects the likelihood of mastitis and lameness in cows. According to the research, the risk of mastitis increases with the cow’s age. This is consistent with the earlier provided information. This indicates that cow age was related to the likelihood of clinical mastitis in the dry period following infusion of internal teat sealant alone at the end of lactation [22]. Age and genetically determined transmitting capacity for lameness were cow-level risk factors for increased lameness prevalence [23]. However, our research revealed no relationship between age and lameness. The few age group categories in the data may be to blame for this outcome. It is necessary to conduct additional research on the impact of age on lameness using more cows of various ages.

There are multiple risk factors that impact clinical mastitis and lameness, including age, nutritional status, perinatal immunity, environmental conditions, and mechanical damage [23, 24]. In our study, we made every effort to minimize the influence of these aforementioned factors by carefully selecting dairy cow samples. However, unavoidable age-related factors were included in our analysis. Nevertheless, apart from age, there may be other unconsidered factors such as diseases caused by mechanical injuries. The main reason for this is the absence of disease records related to veterinarian diagnoses. Although we attempted to mitigate the impact of this factor through frequent visits to the pasture, it remains an area for further improvement in our study.

Zindove et al. (2015) revealed that features like body depth and length impacted stillbirth and ascribed the influence to dietary factors [25]. In contrast to the rump, these traits do not appear to be direct disease sites and may not even be related to reproduction. Therefore, we were more interested in the FL traits directly linked with lameness and the MS and ANG traits directly linked with mastitis in our investigation. To identify a more accurate and direct indicator of disease risk, we examined the impacts of various linear scoring grades of the MS, and ANG traits on clinical mastitis and the effects of different linear scoring grades of FL traits on lameness. Our findings imply that as the RTP trait score increased, the risk of mastitis decreased. The relative positions of the teats determine the scores for this trait; the more the teats converge towards the center, the higher the scores. When cows rest on their sides, this more centered teat phenotype may lead to less contact with the ground. The risk of developing mastitis depends on both the effectiveness of the host immune system and exposure to pathogenic bacteria [26]. The best way to prevent environmental mastitis is to keep the udders clean and reduce teat exposure. The teats of dairy cattle are closely exposed to possible infections in their environment for 12 to 14 h a day while lying down [27].

Additionally, the prevalence of mastitis in cows of various groups was significantly influenced by the FUA, ANG, RAH, and RTP lactation-related traits (*P* < 0.05). According to the findings of logistic regression analysis, the group with high scores of FUA, ANG, and RTP traits had a low risk of onset, whereas the group with low scores of RAH traits had an increased risk of onset. These qualities related to lactation significantly affected the incidence of mastitis. The strength of the foreudder’s attachment to the body’s abdominal wall is reflected in the FUA trait. A higher score for this trait suggests that the muscles securing the foreudder to the body’s abdominal wall are more powerful and tight, indicating a more robust body. According to earlier studies, the strong FUA increased longevity [28–30]. Strong FUA was additionally linked to a lower somatic cell score (SCS) [31]. Less research has been done on how ANG affects the disease. More angular cows are said to perform better during milking [32, 33]. A healthy body is frequently linked to good performance [34]. The distance between the upper breast tissue margin and the lower end of the vulva was used to measure the RAH. This can indicate udders that are drooping. Numerous research studies revealed that udders that are taller and less pendulous in cows have higher mastitis resistance [35]. According to a genome-wide association analysis, the novel quantitative trait loci (QTL) linked to mastitis was found in cows with a loose anterior udder attachment and a low posterior udder height. It might aid in the growth of mastitis [36].

Lameness is the primary health issue causing financial losses and mastitis [37]. The environment, management, and the cows are risk factors of lameness [23]. The cow-level risk variables were high parity, low body condiciton scrore (BCS), the presence of hock injuries, and enlarged claws [38]. Our research discovered that the RLRV substantially impacted the prevalence of lameness. The likelihood of lameness decreases with a narrower angle between the back leg’s tibia and the tarsal bone. A sprain during activity may be prevented by this smaller angle and its potential to increase the stability of the back leg [39]. Previous studies have demonstrated a significant linear relationship between rear limb side view and locomotion in first-lactation daughter locomotion score and/or lameness predictions using sires’ projected breeding values for conformation traits [40].

## Conclusions

In conclusion, our study is the first to establish a significant correlation between body conformation traits observed in early lactation cows and the incidence of clinical mastitis and lameness during lactation. The FUA, RAH, RTP and ANG had a significant impact on the risk of mastitis in dairy cows. The RTP may be an ideal marker for risk of mastitis in cows. These findings may help farmers to identify cows at high risk of diseases in early lactation and give special care to avoid mastitis and lameness. It may also provide guidance for the selection of fertilized cows in the next stage.

## Methods

### Ethics approval

The Yangzhou University Institutional Animal Care and Use Committee reviewed and approved the animal experiment. The “Guidelines for Experimental Animals” of the Ministry of Science and Technology (Beijing, China) were followed when experimenting.

### Animals

In September 2019, 1472 healthy Chinese Holstein milk cows from a well-managed farm in Jiangsu, China, were selected for this study. These cows had comparable traits, including days in milk (30–40 days), milk yield (21–35 kg/day), type of parturition (standard), and a range of ages (2–5 years). Furthermore, no history of calving-related diseases, including metritis, endometritis, hypocalcemia/hypomagnesemia, sub-acute ruminal acidosis, etc.

### Phenotypic score

We rated the body conformation traits of all cow samples based on the industry standard “Code of Practice of type classification in Chinese Holstein” (https://std.samr.gov.cn/gb/gbQuery, Standard No: GBT35568-2017). In summary, 20 linear type traits and six composite traits are among the body conformation traits. The 20 linear type traits were evaluated on a nine-point scale, while the six hybrid traits were measured on an index with values ranging from 40 to 100. When the composite trait score is ≥ 80 points or more, it is considered excellent; a score < 80 points is considered average. Functional scores, weights, and defective traits were the basis for calculating the scores for the six composite traits. Table 1 displays the names and abbreviations for these body conformation traits.

### Disease data

The cow samples were interviewed weekly after evaluating their body conformational traits to gather disease data until they reached the dry milk period. In this study, 802 individual disease records and baseline data were collected. The age was calculated using the fundamental data about the birth date of the cows, and the disease records included the cow number, onset time, and disease content. Next, we obtained the incidence data for mastitis and hoof disease in the disease records that were sourced from the Cattle Farm Veterinary Records. Throughout this period, we only encountered 26 cases of digestive tract diseases apart from these two conditions. It is worth mentioning that all instances of digestive tract diseases occurred more than a month after the onset of clinical mastitis and lameness, thereby having no direct impact on this study. Inaccurate and incomplete information was deleted. The farm veterinarian identified cows with mastitis and hoof diseases. Clinical signs, including abnormal udder (red, swollen, and hard), weird milk, and fever, indicate clinical mastitis [40]. Farm veterinarians diagnose lameness using the visual locomotion scoring method [41]. The diagnosis results of individual diseases determined that the incidence of each disease was “1”, and the incidence of no disease was “0”. Multiple incidences of the same individual are recorded only once.

### Data analysis

Descriptive statistical analysis was performed, and the incidence of lameness and mastitis in dairy cows with various body types was computed. The Chi-square test and Logistic procedure in the Statistical Package for Social Sciences statistical software (SPSS 26.0, IBM, Ehningen, Germany) were used to evaluate the data. The Chi-square test was employed to compare the incidence of clinical mastitis and lameness among cows with varying total score of body conformational traits. Logistic multivariable regression models were constructed to investigate the relationship between body conformational traits and clinical mastitis and claudication development [41–43]. First, factors related to disease development were found by building univariable models (*P* < 0.20). Second, Pearson and Spearman’s correlation coefficients were computed among the significant independent variables to avoid multicollinearity in the following step. Only the independent variable with the highest statistical significance was selected for further analysis when two had correlation coefficients of ≥ 0.55. Ultimately, manual forward stepwise selection was used to construct multivariable models to identify the factors significantly improved the model. Only factors and first-order interactions with *P* < 0.05 were retained in the final model. The age variable was incorporated into the model as a fixed effect, with four levels (2, 3, 4 and 5 years). Additionally, body condition scores ranging from 1 to 9 were in the model as covariates. Using the Hosmer-Lemeshow goodness-of-fit Chi-square test, the model’s fit was evaluated. The direction of the association between the dependent and independent variables was described using the odds ratio (OR). The OR > 1 indicated that the specified amount was more likely to develop clinical mastitis or claudication than the reference value.

## Data Availability

The datasets analysed during the current study are available from the corresponding author upon request.

## Abbreviations

ANG: Angularity
BD: Body depth
BQ: Bone quality
CW: Chest width
TS: Total score
DC: Dairy character
FL: Feet and legs
FA: Foot angle
FTP: Fore teat placement
FUA: Fore udder attachment
FUL: Fore udder length
FC: Frame capacity
HD: Heel depth
LS: Loin strength
MS: Mammary system
MS: Median suspensory
PS: Pin setting
PW: Pin width
RAH: Rear attachment height
RAW: Rear attachment width
RLRV: Rear leg-rear view
RTP: Rear teat placement
RU: Rump
SRL: Set of rear legs
ST: Stature
UD: Udder depth
